# Micro- and Nano-Plastic-Induced Adverse Health Effects on Lungs and Kidneys Linked to Oxidative Stress and Inflammation

**DOI:** 10.3390/life15030392

**Published:** 2025-03-03

**Authors:** Seung Eun Lee, Do Yun Kim, Taek Seung Jeong, Yong Seek Park

**Affiliations:** 1 Department of Microbiology, College of Medicine, Kyung Hee University, Seoul 02447, Republic of Korea; eunlee@khu.ac.kr; 2 Department of Biomedical Science, Graduate School, Kyung Hee University, Seoul 02447, Republic of Korea

**Keywords:** micro-plastic, nano-plastic, environmental contaminant, toxicity, lung disease, kidney diseases

## Abstract

Micro- and nano-plastics (MNPs) are small plastic particles that result from the breakdown of larger plastics. They are widely dispersed in the environment and pose a threat to wildlife and humans. MNPs are present in almost all everyday items, including food, drinks, and household products. Air inhalation can also lead to exposure to MNPs. Research in animals indicates that once MNPs are absorbed, they can spread to various organs, including the liver, spleen, heart, lungs, thymus, reproductive organs, kidneys, and even the brain by crossing the blood–brain barrier. Furthermore, MPs can transport persistent organic pollutants or heavy metals from invertebrates to higher levels in the food chain. When ingested, the additives and monomers that comprise MNPs can disrupt essential biological processes in the human body, thereby leading to disturbances in the endocrine and immune systems. During the 2019 coronavirus (COVID-19) pandemic, there was a significant increase in the global use of polypropylene-based face masks, leading to insufficient waste management and exacerbating plastic pollution. This review examines the existing research on the impact of MNP inhalation on human lung and kidney health based on in vitro and in vivo studies. Over the past decades, a wide range of studies suggest that MNPs can impact both lung and kidney tissues under both healthy and diseased conditions. Therefore, this review emphasizes the need for additional studies employing multi-approach analyses of various associated biomarkers and mechanisms to gain a comprehensive and precise understanding of the impact of MNPs on human health.

## 1. Introduction

Plastic products are used worldwide because of their durability, lightweight nature, resistance to degradation, cost-effectiveness, and excellent insulation properties against heat and electricity. Plastic production has increased significantly, from 1.7 million tons in the 1950s to over 348 million tons in 2017 [1,2]. Currently, there are approximately 45 types of commercially available plastics, including polypropylene (PP), polyethylene (PE), polyethylene terephthalate (PET), polystyrene (PS), polyurethane (PU), polyvinyl chloride (PVC), and polycarbonate (PC) [3]. The extensive use of plastics has caused major environmental challenges, particularly in aquatic ecosystems [4,5,6]. Recent studies have estimated that over 170 trillion plastic particles, weighing 1.1–4.9 million tons, are drifting into oceans worldwide [7].

**Figure 1:**
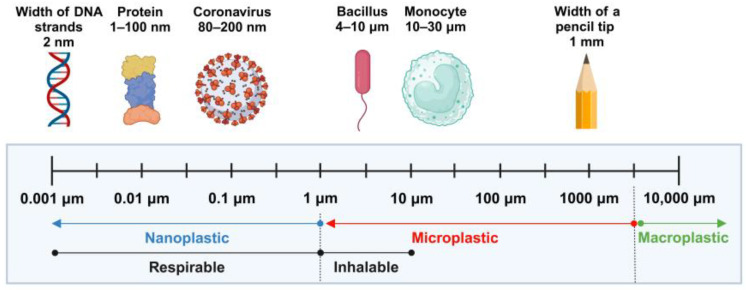
The size of micro- and nano-plastics. Created with BioRender.com. Accessed on 23 December 2024.

Recent studies have focused on the potentially harmful effects of micro-plastics (MPs) on living organisms [8,9]. Once plastic waste is deposited in the environment, it is exposed to ultraviolet (UV) radiation, oxidation, biodegradation, decomposition, and mechanical abrasion. These processes contribute to the fragmentation of plastics into smaller particles, ultimately leading to the formation of micro- and nano-plastics (MNPs) [10]. MPs, with a size range of 1 μm to 5 mm, and nano-plastics (NPs), with a size range of 1 nm to 1 μm, are emerging environmental pollutants associated with plastic waste [11,12] (Figure 1).

**Figure 2:**
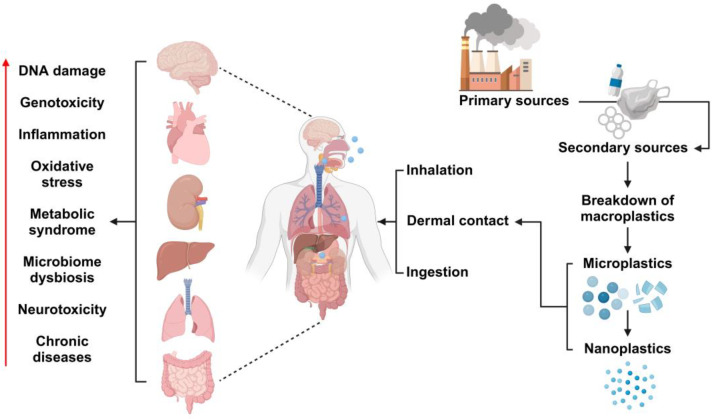
MNP intake and accumulation in vivo. Created with BioRender.com (accessed on 23 December 2024).

MNPs can be classified into two types based on their origin. Primary MNPs originate from plastic pellets, scrubbers, plastic resin flakes, plastic powders, commercial cleaning abrasives, or fluff used in the production of plastic products before entering the manufacturing process [13]. Secondary MNPs, on the other hand, result from the degradation of larger plastic materials [14], with the fragmentation rate varying depending on environmental conditions. MNPs infiltrate the environment through terrestrial sources, atmospheric deposition, airborne transport, textiles, and aquaculture. Additionally, specific degradation processes, including ultraviolet (UV) exposure and bacterial activity, further break down plastic entering the environment into micro- and nano-scale particles [15]. MNPs have become pervasive in ecosystems worldwide, comprising the majority of plastic pollutants [16,17,18]. MNPs, originating from the degradation of plastic materials in food packaging, bottles, and cosmetics due to solar radiation, mechanical fractionation, and weathering processes, enter the human body predominantly through inhalation, ingestion, and dermal absorption. Upon entry via the trachea and esophagus, MNPs accumulate in the lungs and gastrointestinal tract, where they may be internalized into cells through endocytic and phagocytic mechanisms. Furthermore, MNPs can penetrate the circulatory system, transported via blood and lymphatic fluids, leading to their accumulation in critical organs such as cardiac tissue and the brain, posing significant health risks (Figure 2). The size and shape of MPs substantially influence their toxicity. These MPs are composed of various polymers, including additives, colorants, and absorbed chemicals. The chemical composition of MPs can further affect their toxicity; for example, specific polymers may release toxic monomers or degradation products, whereas additives can leach out and produce harmful effects [19]. Therefore, MPs, in addition to their physical toxicity caused by ingestion and their effects on vital organs, exhibit chemical/metal-based toxicity. This further amplifies the harmful potential of MPs.

**Figure 3:**
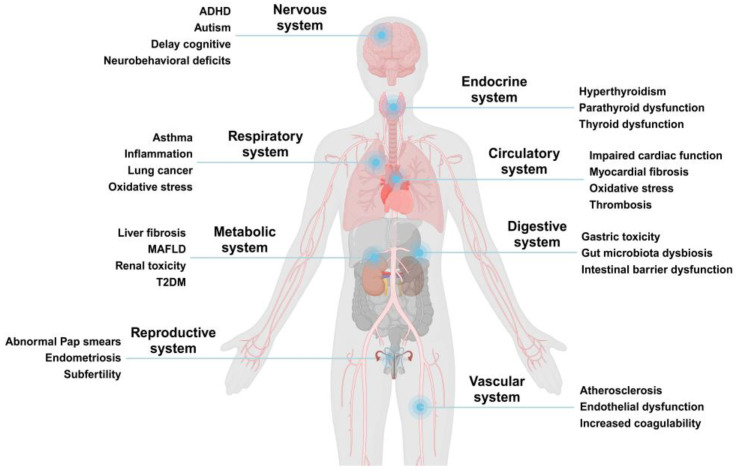
Major systems affected by MNP exposure and related diseases and symptoms. Created with BioRender.com. Accessed on 23 December 2024.

Evidence is limited regarding the potential role of MNPs in human diseases and medical research is still in its infancy in this area. However, an increasing number of in vitro and vivo toxicity studies have highlighted their effects in human cells, primarily linked to oxidative stress and inflammation (Figure 3). This review explores the potential toxicity of MNPs in human organ systems, focusing specifically on the lungs and kidneys. By summarizing the current knowledge, this review aims to provide insights for further research to enhance our understanding of the impact of MNPs on human health.

## 2. MNP-Induced Generation of Reactive Oxygen Species (ROS)

Numerous studies have indicated that oxidative stress can significantly influence damage caused by MNPs [20,21]. In toxicology, oxidative stress encompasses the initial oxidative effects induced by particles and the subsequent formation of ROS in cells or tissues exposed to these particles. Studies in various disease models have shown that ROS generation is a major contributor to oxidative stress and inflammatory responses resulting from MNP exposure [22].

### 2.1. ROS Generation Induced by MNP Exposure

Studies have suggested that the high presence of polystyrene MPs in aquatic environments can damage liver and pancreatic tissues, leading to pancreatic dysfunction and inflammatory responses. This damage may result from a significant increase in ROS levels [23]. PS-MPs cause tissue damage and result in abnormalities in liver and pancreatic function. PS-MPs activate the toll-like receptor 2 (TLR2) signaling pathway and decrease the activity of antioxidant enzymes, such as catalase (CAT), glutathione peroxidase (GSH-Px), and superoxide dismutase (SOD), as well as the total antioxidant capacity (T-AOC), whereas the excessive accumulation of ROS leads to oxidative stress [23]. Exposure of zebrafish (*Danio rerio*) gills to varying concentrations of PS-MPs, specifically 10 and 100 μgL^−1^, resulted in the generation of ROS, which considerably affected oxidative and immune defense mechanisms [24]. This exposure led to significant alterations in the expression profiles of key antioxidant genes, including *cat*, *sod1*, *gpx1a*, and *gstp1*. In addition, PS-MP exposure upregulated pro-apoptotic genes, such as *p53*, *gadd45ba*, and *casp3b*, resulting in increased apoptosis. The oxidative damage caused by PS-MP exposure results in cytological impairments such as disorganization of gill tissue layers, capillary dilation, and necrosis. Exposure to NPs increases the generation of ROS, which affects the activity of antioxidant enzymes such as SOD, CAT, and glutathione peroxidase (GPx) in *Daphnia pulex* [25,26], juvenile *Larimichthys crocea* [27], and *Brachionus koreanus* (monogonont rotifer) [28]. These studies provide several ideas and theoretical foundations regarding the correlation between ROS generation, oxidative stress, and inflammatory response damage induced by MNP exposure in aquatic environments.

Treatment with PS-MPs leads to excessive generation of intracellular ROS in swine testis (ST) cells [29]. This, in turn, promotes the phosphorylation of various genes associated with the mitogen-activated protein kinase (MAPK) pathway, including P38, c-Jun N-terminal kinase, and extracellular signal-regulated kinase, ultimately activating the downstream hypoxia-inducible factor (HIF1α) gene. These findings indicate that treatment with PS-MPs triggers apoptosis and necrosis in ST cells through the ROS/MAPK/HIF1α signaling pathway. Research has indicated that treatment with PS-MPs does not affect the survival rate of myoblasts; however, it increases intracellular ROS production and oxidative stress [30]. This treatment reduced the phosphorylation of p38 MAPK, which inhibited myogenic differentiation, while simultaneously increasing NF-κB expression, thereby promoting adipogenic differentiation. These findings suggest that excessive ROS production caused by PS-MPs disrupts skeletal muscle regeneration and affects the fate of satellite cells in mice. PS-NPs activate the ROS/MAPK signaling pathway, worsening necroptosis and inflammation in mouse spleens [31]. This study demonstrated that PS-NPs contribute to immune dysfunction and inflammatory damage in organisms. Furthermore, a recent study identified that MPs stimulate macrophage inflammation and induce macrophage apoptosis through the upregulation of ROS and the activation of mitochondrial-dependent and mitochondrial-independent pathways [32]. These studies demonstrate that MNP exposure induces ROS generation not only in aquatic environments but also in mammals, leading to oxidative stress, inflammatory response damage, and even cell apoptosis through various pathways. Furthermore, they provide ideas and a theoretical basis for future research.

### 2.2. Oxidase-Mediated ROS Generation Induced by MNP Exposure

It is widely acknowledged that MNPs promote ROS generation and disrupt the capacity of the antioxidant system, thereby inducing oxidative stress [33]. TLRs are essential components of the innate immune system that mediate NADPH oxidase (NOX) activation. The TLR4/NOX2 signaling axis induces ROS generation under various stress conditions [34,35]. Exposure of carp hearts to PS-NPs with particle sizes of 50, 100, and 400 nm acts on the TLR4/NOX2 signaling axis, increasing ROS levels and inducing oxidative stress. These results indicate that exposure to PS-NPs induces oxidative stress, leading to inflammation and apoptosis in carp hearts, with the extent of damage showing a negative correlation with the particle size of PS-NPs [36]. In addition, it causes an imbalance between Th1 and Th2 responses in carp myocardial tissues, leading to inflammatory damage. This process activates the insulin-like growth factor-binding protein 3 (IGFBP3)/p53/acetylcholinesterase (AChE) signaling pathway, ultimately resulting in cardiomyocyte apoptosis. This study highlights the effects of NPs on the heart, emphasizing the relationship between oxidative stress and ROS generation while proposing potential mechanisms.

Wu et al. reported that PS-MPs induce ROS generation, act on the TLR4/NOX2 signaling axis, and activate the Notch and transforming growth factor-β (TGF-β) signaling pathways, causing uterine fibrosis in mice [37]. NPs increase ROS formation in endothelial cells (ECs) [38]. Elevated oxidative stress levels are linked to the induction of NOX expression, which subsequently leads to the downregulation of Sirtuin 1 (Sirt1) expression. The findings indicate that NPs induce premature EC senescence through the redox-sensitive endothelial nitric oxide synthase (eNOS)/Sirt1 signaling pathway. This study emphasizes the effect of NPs on the cardiovascular system and proposes potential underlying mechanisms. These studies present several ideas and theoretical foundations regarding oxidase-mediated ROS generation induced by MNP exposure and its effects, which may be beneficial for future MNP risk assessment.

### 2.3. Mitochondrial Dysfunction-Mediated ROS Generation Induced by MNP Exposure

The disruption of mitochondrial homeostasis and dysfunction primarily occur owing to electron leakage, where molecular oxygen (O_2_) is incompletely reduced to H_2_O in the electron transport chain, leading to ROS generation [39,40]. Mitochondrial dysfunction associated with MNP exposure includes impairment of mitophagy, mitochondrial DNA damage, and excessive ROS production [41]. Pan et al. determined that PS-MPs may be hepatotoxic at environmentally relevant levels, significantly increasing ROS levels and reducing mitochondrial membrane potential (MMP) [42]. Chronic PS-MP exposure induced the disorganization and destruction of the peri-lobular layer owing to the activation of the eukaryotic initiation factor-2 α (eIF2α), activating transcription factor-4 (ATF4) and the C/EBP homologous protein (CHOP) axis. Furthermore, preventing excessive activation of protein kinase R-like endoplasmic reticulum kinase (PERK) may help reduce the cytotoxic effects induced by PS-MPs, which are linked to CHOP signaling and mitophagy changes. This study provides fundamental toxicological data for elucidating the effects of MPs on mammals. Chronic exposure of adult zebrafish to naturally weathered MPs at the highest tested concentration resulted in organ-specific responses, with animals exhibiting anxiety-related disorders and liver-generated ROS species [43]. Mitochondrial damage and subsequent dysfunction caused by the excessive production of ROS have been identified as the primary drivers of the observed effects. Exposure to aged PS-MPs (A-PS) causes oxidative damage because of the increased production of ROS and subsequent DNA damage, leading to a reduction in MMP and the release of cytochrome c (cyt c) from the mitochondria [44]. Activation of the caspase-3/-9 signaling pathways can lead to developmental toxicity through mitochondrial apoptosis. These findings highlight the need to address the environmental impact of aged MPs and emphasize the requirement for further studies to mitigate their potential risks to aquatic ecosystems and human health. This study showed that PS-NPs can enter nonprofessional phagocytes during endocytosis, leading to excessive ROS production [45]. The direct interaction between PS-NPs and intracellular ROS causes substantial damage to autolysosomes and mitochondria, causing MMP depolarization and mitochondrial dysfunction. Consequently, mitochondria-dependent cell death was observed in the larvae and ZF4 cells. These studies emphasize the potential indirect and cumulative environmental impacts of MPs on aquatic ecosystems. Therefore, these studies suggest that future research can focus on ROS generation and mitochondrial dysfunction in response to MNP exposure in aquatic ecosystems.

Di-(2-Ethylhexyl) phthalate (DEHP) and MPs affected neural mitochondria in the mouse brain through the phosphatidylinositol-3-kinase (PI3K)/protein kinase B (AKT) pathway, causing elevated ROS levels and eventually cellular apoptosis [46]. In addition, DEHP/MPs influenced glycogen synthase kinase-3β (GSK-3β) through the PI3K/AKT pathway, disrupting the energy supply to mitochondria. When DEHP and MPs act simultaneously, their combined toxic effects significantly increase neuronal apoptosis in the mouse brain. The results of this study advance our understanding of the neurotoxic effects of DEHP and MPs on rat brain neurons, thus emphasizing the vital role of mitochondrial normalization in safeguarding cerebral health. PS-MPs reduce adenosine triphosphate (ATP) content, decrease MMP, compromise mitochondrial genome integrity, and disrupt the balance between mitochondrial division and fusion in mouse GC-2 cells [47]. This leads to activation of the mitochondrial PTEN-induced putative kinase 1 (PINK1)/Parkin autophagy pathway. Time-course analysis showed that PS-MPs damaged mitochondrial structures through cellular oxidative stress. This study demonstrates the mitochondrial toxicity of PS-MPs and provides a foundation for understanding the mechanisms underlying sperm damage caused by PS-MPs.

Salimi et al. showed that MNPs produce excessive ROS in the cytoplasm, which facilitate the opening of Na/K transmembrane channels in the mitochondrial membrane [48]. This disruption leads to an irregular MMP, causing damage to lysosomes and mitochondria, lipid peroxidation, and depletion of glutathione, all of which contribute to toxicity in human lymphocytes. In addition, the study revealed that human lymphocytes are more susceptible to MNP toxicity than fish lymphocytes. MPs induce mitochondrial dysfunction through NOX4 in various cell models, leading to changes in membrane potential, disruptions in cellular energy metabolism, and the inhibition of mitochondrial respiration [49,50]. MNP exposure affects ROS generation and mitochondrial dysfunction through various pathways in mammals. Therefore, future studies may focus on exploring therapeutic strategies and protective measures against various diseases induced by MNPs based on these aspects.

## 3. MNP-Associated Respiratory Disease

Multiple sources can contribute to the airborne release of MPs, including synthetic fabrics found in clothing, tire wear (particularly from cars and trucks), household products, waste incineration, construction materials, sewage sludge, landfills, abrasive powders, three-dimensional (3D) printing, and the resuspension of polymer particles from urban dust [51,52]. Airborne MP pollution can originate from various sources, such as the leakage of microfibers during washing clothes, which enter the water cycle [53,54]. A small number of MPs in the respiratory tract can stimulate ROS release, resulting in alterations to lung cell metabolism, growth, and cohesion [55]. Recent studies have identified 21 different types of MPs in sputum samples, with polyurethane constituting the majority. These findings indicate that inhalation could serve as a potential entry point for MPs [56]. However, the effect of airborne MPs on public health remains uncertain. Discussion of the potential negative effects of airborne MPs on human health has only recently emerged [57,58].

### 3.1. MNP-Induced Oxidative Stress in Asthma

Recent studies have indicated that inhalation of NPs can cause bronchial epithelial damage owing to epithelial barrier infiltration, which triggers inflammatory responses, cytotoxic effects, and genotoxicity. Prolonged exposure to NPs may result in respiratory diseases such as asthma and pneumoconiosis [59,60,61]. Although several studies have documented the toxicity of MPs, the precise mechanisms through which MP exposure contributes to the development of respiratory diseases are not fully understood.

Based on the 2019 Global Burden of Disease Report, asthma affects approximately 264.2 million individuals worldwide (with a 95% uncertainty interval [UI] of 224.05–309.45 million), with 461,100 deaths (95% UI of 366.58–559.01 thousand), and the number of disability-adjusted life years (DALYs) is 21.5 million (95% UI of 17.14–26.97 million) [62]. Asthma is a complex allergic disease characterized by chronic airway inflammation and oxidative stress [63]. The key immune cells involved in asthma include eosinophils, neutrophils, lymphocytes, and mast cells, which produce ROS and reactive nitrogen species (RNS). These substances act as inflammatory mediators and disrupt the redox balance in the body [64]. The emphasis on the role of ROS in asthma has arisen from the association between environmental pollutants, such as ozone, cigarette smoke, particulate matter (PM), and MNPs, and the increased incidence and severity of asthma [65]. These pollutants often possess oxidative properties, and the inflammatory cells involved in asthma pathophysiology generate ROS. Oxidative stress plays a significant role in the development of asthma. Elevated levels of hydrogen peroxide (H_2_O_2_) have been observed in the exhaled breath of patients with asthma compared with healthy individuals, and higher concentrations of H_2_O_2_ correlate with the severity of the condition [66]. Macrophages isolated from the airways of patients with asthma produce large amounts of superoxide anions, and eosinophils exhibit increased ROS generation upon antigen stimulation [67,68]. The ratio of oxidized peroxiredoxins to total peroxiredoxins in peripheral blood mononuclear cells from patients with asthma is higher than in healthy individuals, and more oxygen radicals are generated following H_2_O_2_ treatment [69]. These findings indicate that the peroxiredoxin activity responsible for managing free radicals in patients with asthma is reduced compared with healthy individuals, leaving them more vulnerable to oxidative stress.

Although the relationship between air pollution and the development of asthma has been well-documented [70,71], the potential impact of MNPs on the onset and severity of asthma remains insufficiently explored. However, the condition described as “meat-wrapper’s asthma” in women cutting PVC suggests that high doses of inhaled plastics and/or leached chemicals may trigger asthma-like symptoms [72,73]. Chen et al. compared the levels of MPs in bronchoalveolar lavage fluid (BALF) between children with community-acquired pneumonia and those with asthma [74]. Higher levels of MPs were detected in the BALF of severe cases of community-acquired pneumonia than in mild cases; however, no significant differences were found between community-acquired pneumonia and asthma patients. In contrast, lower levels of MPs were recorded in the nasal lavage fluid of healthy volunteers compared with that in patients with allergic rhinitis, a condition commonly linked to allergic asthma [75]. These findings highlight the potential impact of MNPs on the onset and severity of asthma and may help elucidate the underlying mechanisms of airway pathology induced by MNP exposure.

In animal studies, the exposure of mice to amino-resin spheres resulted in a significant increase in eosinophil and lymphocyte counts in BALF compared with the control groups [76]. Furthermore, a notable increase in the number of macrophages in lung tissue was observed. In house dust mite-treated mice, a model for allergic asthma, the concurrent inhalation of MPs led to a more pronounced increase in the number of macrophages and enhanced mucus production [76]. In another model of allergic asthma using ovalbumin-treated animals, the ingestion of PS-MPs exacerbated traits (airway inflammation and hyper-responsiveness) associated with allergic asthma [77]. Co-exposure to PS-MPs and DEHP caused significantly greater damage, airway mucus hypersecretion, an imbalance between Th1 and Th2 responses, and elevated levels of oxidative stress through activation of the transient receptor potential ankyrin 1 (TRPA1) and p38 MAPK pathways.

Occupational and animal exposure may indicate that MNPs or their leaching components contribute to the characteristics associated with allergy-related asthma. Therefore, exposure to high levels of MNPs, commonly found indoors, can exacerbate asthma symptoms and trigger exacerbations. These findings suggest that future studies may focus on MNP exposure and oxidative stress in the onset and severity of asthma.

### 3.2. MNP-Induced Oxidative Stress in Chronic Obstructive Pulmonary Disease (COPD)

COPD is a progressive and chronic condition characterized by irreversible airflow limitation and chronic bronchitis and/or emphysema and is associated with a progressive decline in lung function [78]. COPD can develop as a result of genetic risk factors or prolonged exposure to irritants such as cigarette smoke, toxic gases, PM, and MNPs. Based on the 2019 Global Burden of Disease Report, the global mortality rate for COPD was 35.4%, and the prevalence was 222.3 million (95% UI of 200.4–225.1) [79]. The disease resulted in approximately 3.3 million deaths (95% UI of 2.9–3.5 million) and 74.4 million DALYs (95% UI of 68.2–80.1 million). Redox imbalance may serve as an additional mechanism through which MPs influence chronic respiratory diseases [80]. Oxidative stress arising from this imbalance is a significant factor contributing to the pathogenesis of various pulmonary conditions, including asthma, COPD, and acute respiratory distress syndrome (ARDS) [65]. Studies have indicated that exposure to MPs derived from the environment leads to a reduction in antioxidants within simulated lung fluid [81].

Information regarding the potential link between MNPs and COPD onset and progression is limited. Regarding smoking, which is considered a major risk factor for the development of COPD, Lu et al. recently identified MPs ranging from 20–500 µm in cigarette smoke [82]. The same study indicated that smokers had a higher quantity and greater diversity of MPs in their bronchoalveolar lavage fluid than non-smokers.

Two studies examined the direct effects of MNPs on COPD parameters in lung epithelial cells. In a bronchial epithelial cell line, exposure to PS-MPs led to a decrease in the expression of the α1-antitrypsin protein, which is a well-established risk factor for the development of COPD [83]. Similar effects were observed in a lung-on-a-chip model following the introduction of PS-NPs [84]. Jin et al. determined that four types of MPs increased ROS levels in human lung-derived cells and BEAS-2B cells, effectively inducing cellular aging in human lung epithelial cells [85]. This study demonstrated that MPs activate ROS signaling, leading to aging in both human lung epithelial cells and mouse lungs, thereby offering novel insights into the potential role of MPs in lung diseases. Therefore, future studies should focus on exploring the impact of MPs on pulmonary diseases, particularly the role of oxidative stress and its mediated effects.

In a mouse model exploring the oronasal aspiration of PS-NPs, PS-NPs were observed to accumulate in the lungs and affect lung organ counts [86]. Furthermore, PS-NPs induced both localized and systemic oxidative stress, inflammation, and an imbalance of protease–antiprotease activity, ultimately leading to reduced respiratory function and lesions resembling those observed in COPD. In addition, PS-NPs may activate intracellular mechanisms, including mitochondrial dysfunction and ER stress, which may contribute to COPD progression. This study provided a comprehensive and systematic examination of the detrimental effects of PS-NPs on respiratory health, highlighting their potential role in increasing the risk of COPD.

In a mouse model subjected to chronic exposure to PS-MPs through intratracheal instillation, PS-MPs increased collagen fiber deposition, decreased lung tissue barrier permeability, and impaired lung function [87]. Moreover, following the inhalation of PS-MPs, there was a notable increase in the presence of Gram-negative bacteria within the pulmonary flora, which triggered the release of lipopolysaccharides (LPS) and subsequently increased the expression of its primary receptor, TLR4. This cascade of events ultimately caused ferroptosis in lung tissue cells. Further in vitro intervention experiments indicated that the disruption of lung iron homeostasis, driven by the pulmonary microbiota/TLR4 pathway, is a crucial mechanism underlying lung damage associated with PS-MPs. These data provide new evidence of lung injury induced by environmentally relevant levels of MPs and suggest prevention strategies based on long-term dynamic observations.

### 3.3. MNP-Induced Oxidative Stress in Pulmonary Fibrosis

Pulmonary fibrosis is defined by the abnormal degradation and remodeling of the extracellular matrix, resulting in the excessive accumulation of collagen, fibrin, and other matrix components and causing irreversible damage to the distal lungs [88]. Pathological processes of this nature interfere with normal gas exchange, resulting in pulmonary dysfunction and failure [89,90]. Oxidative stress resulting from an imbalance between ROS and RNS leads to cellular dysfunction and damage to lung epithelial tissues, thereby promoting fibrosis [91]. Furthermore, cytokines and growth factors released by lung inflammatory cells such as macrophages and neutrophils produce elevated levels of ROS and RNS, which further exacerbate the fibrotic process. Several studies have indicated that exposure to PVC dust can induce pneumoconiosis with a granulomatous response of macrophages to PVC particles, potentially resulting in interstitial fibrosis in humans [92,93,94]. In addition, PVC monomers have been shown to cause pulmonary fibrosis by interacting with proteins and triggering an immune response against these altered proteins [95].

The risk assessment of tire wear micro-plastic particle (TWMP) inhalation suggests that miR-1a-3p targets twinfilin-1, a protein that regulates the cytoskeleton [96]. This inhibits the formation of F-actin and disrupts cytoskeletal rearrangement, potentially leading to pulmonary fibrosis following TWMP exposure. A notable characteristic of pulmonary fibrosis is the abnormal accumulation of collagen, particularly collagen type Iα1 (COL1A1). Sirius red staining of lung tissue from mice exposed to TWMPs showed collagen deposition, indicating the potential development of pulmonary fibrosis. These findings highlight the mechanism by which TWMP induces pulmonary fibrosis through miRNA-mediated cascade reactions and may provide a theoretical basis for the role of epigenetics in lung injury.

The long-term inhalation of PS-MPs causes pulmonary toxicity and fibrosis [97]. Exposure to PS-MPs significantly reduced glutathione (GSH) levels and increased malondialdehyde (MDA) levels, leading to iron overload in both mouse models and alveolar epithelial cells (AEC) in lung tissues. In addition, PS-MPs promote cellular ferroptosis and activate the cyclic GMP-AMP synthase (cGAS)/stimulator of interferon genes (STING) signaling pathway, thereby contributing to the progression of pulmonary fibrosis. These findings provide valuable insights into the mechanisms underlying PS-MP-induced pulmonary fibrosis and suggest potential therapeutic strategies.

This study showed that the inhalation of PS-MPs was linked to pulmonary fibrosis [98]. Exposure to PS-MPs causes oxidative stress and damage to the alveolar epithelial cells, as observed using a microscope. These damaged cells activate pulmonary fibrosis through the Wnt/β-catenin signaling pathway. These findings offer new insights into the potential risks associated with PS-MP inhalation in terrestrial mammals. These studies highlight the impact of MP exposure on pulmonary fibrosis and its association with oxidative stress, providing potential mechanisms. They suggest that future studies may focus on these aspects.

## 4. MNP-Associated Renal Disease

With increasing concerns regarding the impact of MPs on human health, a surge in research involving human tissues and cells has occurred in the medical community. This trend extends to nephrology. However, definitive evidence regarding the effects of MPs and their compounds on kidney cells and tissues remains insufficient. Recent research on the renal toxicity of MNPs has gained momentum, and most studies have been published in the past three years. Li et al. demonstrated that polystyrene nano-particles (PS-NPs) exacerbated cell death in rat kidney cells by inducing oxidative stress through the endoplasmic reticulum (ER) stress pathway triggered by LPS [99]. Furthermore, Tang et al. reported that kidney toxicity in rats was linked to inflammation, oxidative stress, and lipid disruption [100]. After 6 weeks of exposure to PS-NPs, the rats showed a decrease in the kidney index, along with signs of tubular atrophy, glomerular collapse, and infiltration of inflammatory cells, resulting in impaired kidney function. Although some studies have assessed both NPs and MPs, their distinct sizes lead to varied characteristics. The formation of a protein corona in biological solutions, along with its absorption and uptake by cells and tissues, differs significantly between NPs and MPs, complicating direct comparisons of their effects in vivo and in vitro.

A pioneering study by Deng et al. (2017) explored the accumulation and toxicity of PS-MPs in mice using fluorescent microspheres [101]. These findings revealed that MPs were deposited in kidney tissues and induced metabolic changes, including oxidative stress and alterations in energy and lipid metabolism, as determined by metabolic analysis.

The accumulation of MPs in the kidneys of mice and their absorption through HK2 have been confirmed, revealing that PS-MPs lead to increased mitochondrial ROS, elevated levels of ER stress-related proteins, and markers of inflammation and autophagy [102]. Meng et al. reported that the exposure of mice to PS-NPs and PS-MPs resulted in a significant increase in SOD and GSH-Px levels, along with a marked inhibition of CAT [103]. These findings provide evidence of the detrimental effects of PS-MPs on mouse kidneys through oxidative stress and inflammation. Collectively, these studies reveal the distribution and accumulation of MPs in mice kidney tissue and provide a theoretical basis for the association between oxidative stress and significant changes in various biomarkers indicative of potential toxicity induced by MP exposure.

A previous study investigated the effect of PS-MP exposure on kidney tissue damage in chickens over a 6-week duration [104]. These findings indicate that PS-MP exposure causes alterations in mitochondrial morphology and dysbiosis (mitofusin (MFN)1/2, optic atrophy protein 1 (Opa1), and dynamin-related protein (Drp1)), leading to disruptions in mitochondrial dynamics and structural damage. Furthermore, the activity of several antioxidant enzymes (SOD, CAT, MDA, GSH, and T-AOC) was significantly modified, contributing to oxidative stress and necrosis through the activation of the receptor-interacting protein kinase-1 and -3/mixed lineage kinase domain-like protein (RIP1/RIP3/MLKL) signaling pathway. In conclusion, to the best of our knowledge, this study is the first to demonstrate that the oral ingestion of PS-MPs induces inflammation and necrosis in chicken kidneys, with the severity of damage corresponding to the concentration of PS-MPs. These findings offer valuable theoretical insights into the environmental risk of PS-MPs.

Goodman demonstrated the activation of ROS by PS-MPs in human embryonic kidney cells (HEK293) [105]. This study showed that the expression levels of antioxidant markers, including SOD2, CAT, and glyceraldehyde 3-phosphate dehydrogenase (GAPDH), decreased, whereas ROS levels increased. This indicates a reduction in enzymatic activity accompanied by morphological changes. These findings suggested that the capacity of HEK293 cells to mitigate ROS production was compromised, ultimately affecting the overall functionality of kidney cells. This study suggests that the ingestion of micro-plastics may induce toxicity in human kidney cells as an adverse effect of PS-MPs.

The investigation into the renal toxicity of DEHP and PS-MPs in mice and HEK293 cells exhibited a notable increase in the expression levels of genes related to the ROS/AMP-activated protein kinase (AMPK)/Unc-51-like autophagy-activated kinase (ULK1) and Ppargc1α/Mfn2 signaling pathways [106]. In addition, both mRNA and protein levels of autophagy markers were significantly upregulated. In conclusion, exposure to both DEHP and PS-MPs resulted in the excessive production of ROS, leading to oxidative stress and activation of the AMPK/ULK1 pathway, which subsequently triggered renal autophagy. These findings have advanced the understanding of nephrotoxicity associated with plasticizers and MPs, offering new insights into the combined toxicity of DEHP and PS-MPs.

A mouse kidney injury model revealed the harmful effects of MPs and cadmium on kidney function [107]. These effects are mediated by various mechanisms, including oxidative stress, autophagy, apoptosis, and fibrosis. Notably, 5 µm MPs were observed to adsorb cadmium, which triggered notable biological responses such as a reduction in the activity of antioxidant enzymes, such as SOD and CAT, accompanied by an increase in the expression of autophagy markers such as microtubule-associated protein 1 light chain 3 (LC3)-II (LC3-II), as well as early autophagy markers autophagy-related gene 5 (ATG5), ATG7, and beclin-1. Furthermore, there was an upregulation of renal fibrosis markers, including α-smooth muscle actin (α-SMA), TGF-β1, and the type IV collagen alpha chain (COL4A), ultimately leading to alterations in kidney tissue structure and nephrotoxicity. This study demonstrated that particles could enter the systemic circulation, accumulate in the kidneys of mice, and induce severe biological responses. Additionally, it suggests that the effects of plastic particle exposure and Cd contamination are complex, with varying toxic effects across species, providing evidence of the potential threats posed by MPs and their adsorbed heavy metals.

Chronic exposure to MPs in mouse models caused kidney damage [108]. These effects vary depending on the diameter of the MPs, which can lead to oxidative stress, inflammation, and kidney fibrosis. These factors contribute to kidney dysfunction, histological changes, and various pathological states in the kidneys. In addition, transcriptomic data indicate that long-term exposure to MPs can lead to changes in the expression of multiple genes associated with immune responses and circadian rhythms. This study demonstrated that chronic MP exposure induces kidney damage through different mechanisms depending on MP size, suggesting that MPs are a significant risk factor for kidney disease.

Kuang et al. investigated the mechanism underlying the toxic effects of MPs on kidneys using a mouse model of ischemia-reperfusion (IR) [109]. Long-term exposure to MPs can damage the renal tubules and glomeruli, promote inflammation, and contribute to kidney fibrosis, ultimately resulting in kidney dysfunction and histological alterations. This damage became even more pronounced when paired with IR injury models. Pyroptosis, triggered by inflammation, may play a pivotal role in this mechanism. Specifically, MPs activate the NACHT, leucine-rich repeat, and pyrin domain-containing protein 3 (NLRP3) inflammasome and caspase-1 through apoptosis-associated speck-like protein containing a CARD (ASC), which facilitates the cleavage of interleukin-1β (IL-1β) and IL-18, thereby instigating pyroptosis. This process further influences the progression of kidney fibrosis and inflammation, potentially worsening the kidney damage caused by IR. These findings provide valuable insights into kidney damage associated with long-term exposure to MPs in conjunction with IR, establishing a basis for understanding the significance of MPs in human health and disease susceptibility, particularly in vulnerable populations such as those experiencing acute kidney injury (AKI).

Using a mouse model, a previous study explored the toxic effects of various types of PS-MPs, including unmodified PS, negatively charged PS-SO3H, and positively charged PS-NH2 MPs, on the kidneys [110]. The results demonstrate that exposure to these MPs significantly increased the levels of urea, blood urea nitrogen (BUN), creatinine (CREA), and uric acid (UA) in both urine and serum. In addition, white blood cell count and protein levels increased, suggesting kidney dysfunction. This study further revealed that MPs induce chronic inflammation and kidney fibrosis, which are associated with the aging of tubular epithelial cells. Moreover, the research emphasizes the role of the Clotho/Wnt/β-catenin signaling pathway in promoting epithelial–mesenchymal transition (EMT) in tubular epithelial cells, which contributes to kidney fibrosis and dysfunction. These findings highlight the potential risks of long-term MP exposure and the mechanisms underlying kidney damage. Furthermore, it suggests that the expression of target genes induced by MPs, as well as cellular senescence and renal fibrosis, could be key focal points in developing new strategies for disease mitigation.

This study investigated the effects of PS-MPs on human tubular cells and fibroblasts [111]. The results revealed that PS-MPs significantly enhanced the production of extracellular vesicles (EVs) in human tubular cells. Notably, although the PS-MPs did not trigger the expression of inflammation-related proteins in these cells, they stimulated the expression of proteins associated with ER stress. These findings indicated that PS-MPs may have subtle yet significant effects on cellular stress mechanisms, particularly by promoting ER stress and EV production. This can potentially influence intercellular communication and contribute to kidney pathology.

This study investigated the nephrotoxicity associated with MPs, specifically focusing on PS-MPs and amino-functionalized polystyrene (PS-NH2) MPs [112]. Using a mouse model, researchers discovered that chronic long-term exposure to MPs results in kidney fibrosis. This effect is primarily associated with the accumulation of inflammatory cells, which in turn trigger inflammatory responses that contribute to renal tissue remodeling and fibrosis. These findings highlight the potential long-term effects of MP exposure on kidney health and emphasize the significant role of inflammatory processes in MP-induced nephrotoxicity.

Several studies have highlighted the potential toxicity of MNPs and their constituent compounds in human cells and tissues through various mechanisms. Although evidence concerning their effects on human kidneys is still not fully established, both in vitro and in vivo studies suggest that MNPs may pose a risk to renal cells through mechanisms such as oxidative stress, inflammation, and fibrosis.

## 5. Conclusions

In recent years, scientists have investigated the various detrimental effects of MNPs, primarily those derived from PS, on diverse biological systems and models, including cell lines and in vivo animal studies. Evidence gathered from previous studies indicates that prolonged exposure to MNPs can generate ROS and activate cellular inflammatory signaling pathways, potentially resulting in severe adverse effects. Extensive research is required to fully comprehend the implications of MNPs and devise strategies to mitigate their accumulation in ecosystems.

In recent years, numerous physical and chemical approaches have been reported for the removal of pollutants from aqueous solutions. Physical methods, including adsorption, precipitation, distillation, and filtration, as well as chemical methods such as chemical precipitation, coagulation, chemical oxidation, solvent extraction, and ion exchange, are generally more suitable for the removal of bulk or large-scale pollutants rather than micro/nano-pollutants in aquatic environments. Consequently, nanotechnology-based pollution mitigation methods utilizing nano-adsorbents, such as magnetic nanomaterials, carbon nanotubes (CNTs), and metal-organic frameworks (MOFs), have emerged as unique and sustainable solutions for achieving an environmentally friendly approach [113,114,115]. Furthermore, among plastic waste, biodegradable plastics have garnered public attention as a promising alternative to conventional non-degradable plastic polymers, which contribute to severe plastic pollution. Due to their susceptibility to microbial degradation, biodegradable plastics are considered environmentally benign, making them a viable solution for mitigating plastic pollution [116,117,118].

To enhance our understanding of how MNPs contribute to the development and maintenance of diseases affecting the human lungs, kidneys, and other tissues, diverse epidemiological studies are crucial. Additional research is required to standardize the collection, handling, and preparation protocols for human biological samples. Future investigations should also explore the effects of MNPs in combination with other toxic substances and examine whether synergistic toxicity arises from interactions between MNPs and critical additives used in the plastics industry.

This review provides a comprehensive overview of the current understanding of the effects of MNPs on the structure and function of the lungs and kidneys, as well as their influence on the pathophysiology of diseases and disorders.

## Data Availability

Not applicable.
